# Lipid Oxidation Changes of Arabica Green Coffee Beans during Accelerated Storage with Different Packaging Types

**DOI:** 10.3390/foods11193040

**Published:** 2022-09-30

**Authors:** Sai Aung Moon, Sirirung Wongsakul, Hiroaki Kitazawa, Rattapon Saengrayap

**Affiliations:** 1 School of Agro-Industry, Mae Fah Luang University, Chiang Rai 57100, Thailand; 2 Coffee Quality Research Group, Mae Fah Luang University, Chiang Rai 57100, Thailand; 3 Tea and Coffee Institute, Mae Fah Luang University, Chiang Rai 57100, Thailand; 4 Institute of Food Research, National Agriculture and Food Research Organization (NARO), 2-1-12 Kannondai, Tsukuba 305-8642, Ibaraki, Japan; 5 Integrated AriTech Ecosystems Research Group, Mae Fah Luang University, Chiang Rai 57100, Thailand

**Keywords:** green coffee beans, packaging, quality, rancidity, shelf life

## Abstract

The storage conditions of green coffee beans (GCBs) are indispensable in preserving their commercial value. In Thailand, coffee farmers and roasters typically store GCBs for six months to a year before roasting. However, the beans undergo oxidation during storage, influencing both quality and taste. This study investigated changes in GCB lipid oxidation under different accelerated storage conditions (30 °C, 40 °C and 50 °C with 50% RH) and packaging, i.e., plastic woven (PW), low-density polyethylene (LDPE) and hermetic/GrainPro^®^ (GP) bags. Samples were collected every five days (0, 5, 10, 15 and 20 days) and analyzed for lipid oxidation parameters including acid value (AV), free fatty acids (FFA), peroxide value (PV), ρ-anisidine value (PAV), total oxidation value (TOTOX), thiobarbituric acid reactive substances (TBARS), moisture content (MC), water activity (a_w_) and color. Primary oxidation was observed, with AV, FFA and PAV gradually changing during storage from 1.49 ± 0.32 to 3.7 ± 0.83 mg KOH/g oil, 3.82 ± 0.83 to 9.51 ± 1.09 mg KOH/g oil and 0.99 ± 0.03 to 1.79 ± 0.14, respectively. Secondary oxidation changes as PV and TBARS were reported at 0.86 ± 0.12 to 3.63 ± 0.10 meq/kg oil and 6.76 ± 2.27 to 35.26 ± 0.37 MDA/kg oil, respectively, affecting the flavor and odor of GCBs. Higher storage temperature significantly influenced a lower GCB quality. GP bags maintained higher GCB quality than LDPE and PW bags. Results provided scientific evidence of the packaging impact on oxidation for GCB under accelerated storage.

## 1. Introduction

Coffee is now one of the most popular drinks and is widely consumed daily. Arabica varieties are the most productive, have the highest quality, and are grown at high elevations above 1000 m worldwide [1,2]. In 1849, Arabica coffee (*Coffea arabica*) was introduced to Thailand as Catimor, Caturra, Typica, Bourbon, Catuai, Mundo Novo and others [3,4]. The Chiang Rai region of Northern Thailand including Doi Chang, Pang Khon, Mae Chang Tai, Doi Tung and Chiang Mai produces Arabica coffee, with 19,900 ha of farming land mainly located in the highlands [5]. Thai coffee has spread to countries including the United States, Canada, the United Kingdom, South Korea, Singapore, Malaysia, Cambodia and Laos. Thai Arabica coffee has a unique profile of intense fruity-floral aroma, acidity, clean-cup, full profile and flavors depending on the processing method [6,7].

However, farmers have faced unstable coffee bean prices, with a lack of market access and the high production costs of hired labor and chemical fertilizers. Sustainable farming practices are required to support local communities and improve farmers’ living standards [7]. Chuqian [8] also suggested the utilization of advanced processing techniques and equipment to develop a qualified, precise, commercialized industry on a large scale, improve product quality, and conduct more comprehensive investigations and research on problem issues. In Thailand, coffee farmers and roasters typically store green coffee beans (GCB) for six months to a year before roasting. During postharvest and storage, Thai Arabica coffee can be contaminated by fungi that produce mycotoxins such as ochratoxin A (OTA), Aspergillus and Penicillium, particularly on coffee cherries, parchment coffee and green coffee beans [9]. Fungi and mold oxidation reactions are found in the coffee processing steps of fermentation, drying and storage, and these microbiota can affect the final taste and sensory qualities of the product. The packaging bags also impact the quality of green coffee beans. Abreu et al. [10] stated that no packaging system can preserve the initial sensory quality of coffee over long-term storage. This highlights the importance of developing methods to detect and minimize physical, chemical and oxidation changes in green coffee beans during storage before they negatively impact sensory quality [11,12,13,14].

It is necessary to maintain and safeguard GCB quality during long-term storage before roasting to maximize market price [15,16]. Several compounds that give coffee its sensory characteristics can be produced or degraded during storage [14], and effective methods for preserving the sensory quality of coffee beans during storage are vital [11]. Long-term storage and environmental factors alter GCB physical and chemical properties [15]. Molds develop and produce toxins that are harmful to consumer health, reducing production quality and impacting market value and human safety [9]. Scheidig, Czerny and Schieberle [17] stated that coffee bean storage can affect moisture content, density, odor and aroma, all of which influence flavor. The physicochemical properties of coffee quality, aroma, flavor and taste profiles depend on the variety, plantation environment and farming method involving soil type, climate, husbandry (farming), geography (latitude and altitude), harvesting time, processing, storage, packaging and roasting [18,19,20,21,22].

Accelerated storage techniques are commonly used to control the storage environment by reducing the experimental time of long-term assessments [23]. This allows fast aging of food products. The most commonly used accelerating factors are temperature, humidity and light [24,25]. Accelerated storage focuses on the sensory, chemical, biochemical and physical changes [24] as very effective tools for studying the changing characteristics of agricultural products and reduction in coffee quality. Cong et al. [25] noted that lipid oxidation impacts the loss of Robusta coffee quality during accelerated storage. Green coffee beans have low moisture content and a high proportion of unsaturated fatty acids. This enhances lipid oxidation that leads to rancid odors, negatively impacting nutritional quality and product secondary oxidation as harmful to human health [25]. Coffee has high total lipid content of 50%, comprised of unsaturated fatty acid content higher than saturated fatty acids that easily oxidize, with loss of cellular structure, seed viability and sensory changes. The TBARS value, carbonyl groups and caffeoylquinic acid concentration reduce with loss of color during GCB storage [16]. Speer and Kolling-Speer [26] reported that during storage, GCB sensory attributes changed, with production of active lipases, free unsaturated fatty acids and hydroperoxides associated with lipid oxidation. However, scant research has been conducted on how accelerated storage and packaging impact Thai Arabica GCB quality.

Thus, here, changes in moisture content, water activity, color, fatty acids and lipid oxidation as well as acid value (AV), free fatty acids (FFA), peroxide value (PV), ρ-anisidine value (PAV), total oxidation value (TOTOX), thiobarbituric acid reactive substances (TBARS) in GCB under different accelerated storage conditions were identified. The impact of packaging types, i.e., plastic woven (PW) bags, low-density polyethylene (LDPE bags) and hermetic/GrainPro^®^ (GP) bags on the loss of GCB quality was also assessed.

## 2. Materials and Methods

### 2.1. Sample Preparation

Coffee cherries (*Caffea arabica* L.) for this research were collected from Doi Thep Sadet, Chiang Mai, Thailand during the 2021–2022 coffee cherry harvesting season. Coffee cherries were controlled in the fully ripe stage and processed by washing. The samples were de-pulped to remove the outer skin, the parchment was soaked until pH reached 4.3–4.5 and the mucilage was cleaned using clean water. The cleaned coffee parchment was dried using a shred drying device until the moisture content was 10–12%. After that, the coffee samples were de-hulled to obtain GCBs and transferred to the laboratory for further analysis.

**Figure 1:**
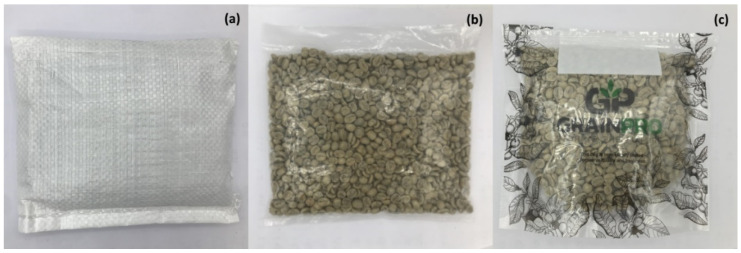
Packages for GCB storage (**a**) plastic woven bag (PW), (**b**) low-density polyethylene bag (LDPE) and (**c**) GrainPro^®^ (GP).

Samples of 250 g of green coffee beans were packed in plastic woven (PW), low-density polyethylene (LDPE) and GrainPro^®^ (GP) bags (Figure 1a–c) and stored in a Constant Climate Chamber (HPP750, Memmert GmbH, Schwabach, Germany) with temperature controlled at 30, 40 and 50 °C and relative humidity (RH) 50%. The properties of packages are shown in Table 1. Air permeability was determined using an air permeability tester (FX 3300 LabAir IV, Textest Instruments, Schwerzenbach, Switzerland) according to ASTM D737-04 [27]. The packages were cut into 20 × 20 cm^2^ and results were reported in L/m^2^ s. The thickness of packages was measured using the thickness gauge (SMD-565J, Teclock, Nagano, Japan). Three replicates were tested and average values were reported. Coffee samples were taken from the accelerated storage chamber every five days (0, 5, 10, 15 and 20 days), repackaged in polyethylene vacuum sealed packages and stored at −80 °C in an ultra-low temperature freezer (MDF-193, SANYO, Tokyo, Japan) until future analysis. Day 0 samples (the control sample) were prepared without any package and used to determine the initial quality parameters of the GCB.

### 2.2. Moisture Content (MC)

Moisture content was measured by drying in a hot air oven at 70 ± 1 °C for 16.0 ± 0.5 h until constant weight [28] (AOAC 2000 method 979.12) and calculated using Equation (1):

(1) $$\%\;\mathrm{Moisture}=\frac{\mathrm{Weight}\;\mathrm{loss}\;\mathrm{on}\;\mathrm{drying}\;\left(g\right)}{\mathrm{Weight}\;\mathrm{of}\;\mathrm{sample}\;\left(g\right)}\times 100$$

### 2.3. Water Activity (a_w_)

Water activity (a_w_) was determined using a water activity meter (Aqua Lab, Decagon, WA, USA) at 25 °C with autoanalysis on ground GCB samples [29].

### 2.4. Color Determination

Color values of GCBs were analyzed using a colorimeter (Color Quest XE, Hunter Associates, Reston, VA, USA) and reported based on the CIELab color scale [30]. The colorimeter was calibrated with a standard white tile to obtain the coordinates for the illuminant D65: L* = 94.64, a* = −0.80 and b* = 0.07. L* measures lightness (L* = 100 means white, L* = 0 means black), a* indicates the contribution of red or green (redness (+) and greenness (−)) and b* determines the role of blue or yellow (yellowness (+) and blueness (−)).

### 2.5. Lipid Oxidation Determination

Lipid oxidation was determined as lipid, fatty acid and primary oxidation including acid value (AV), free fatty acids (FFA), peroxide value (PV) and secondary oxidation as ρ-anisidine value (PAV) and thiobarbituric acid reactive substances (TBARS).

#### 2.5.1. Lipid Content

The lipid content was determined by constant extraction in a Soxhlet apparatus at 105 °C for 2 h using petroleum ether as the solvent [31] (AOAC, 2000 method number 920.39). The lipid content (%fat) was calculated using Equations (2) and (3):

(2) $$\%\mathrm{Fat}\;\left(\mathrm{wwb}\right)=\frac{\left(\mathrm{Weight}\;\mathrm{of}\;\mathrm{can}\;\left(g\right)+\;\mathrm{fat}\right)-\mathrm{Weight}\;\mathrm{of}\;\mathrm{can}\;}{\mathrm{Weight}\;\mathrm{of}\;\mathrm{sample}\left(g\right)}\times 100$$

(3) $$\%\mathrm{Fat}\;\left(\mathrm{dwb}\right)=\frac{\%\;\mathrm{Fat}\;\left(\mathrm{wwb}\right)}{\left(100-\%\mathrm{moisture}\right)}\times 10$$

#### 2.5.2. Determination of Acid Value (AV) and Free Fatty Acid (FFA)

The AV and FFA were measured by titration following the AOAC method number 940.28 [32]. In brief, 1 g of oil sample was weighed into a 250 mL flask and heated to melting point at not more than 10 °C. Then, 50 mL of neutralized alcohol (1:1 *v*/*v*) was added and the mixture was boiled in a boiling water bath until a clear solution was obtained. While still hot, the sample was titrated with 0.1 M potassium hydroxide (KOH) with vigorous shaking until a pink color persisted for 30 s. AV was expressed as milligrams of KOH required to neutralize the FFA present in 1 g of the oil samples (mg KOH/g oil). The AV and FFA were calculated using Equations (4) and (5):

(4) Acid value = mg KOH required per gram of oil (1 mg of 0.1 KOH =5.6 mg KOH)

(5) $$\mathrm{Free}\;\mathrm{fatty}\;\mathrm{acids}\;(\mathrm{as}\;\mathrm{palmitic}\;\mathrm{acid}),\;\%=\frac{Volume\;\mathrm{KOH}\times Normality\;\mathrm{KOH}}{\mathrm{weight}\;\mathrm{of}\;\mathrm{oil}}\times 25.6$$

#### 2.5.3. Determination ρ-Anisidine Value (PAV)

The PAV was measured according to Cong et al. [25]. The oil sample (0.6 g) was placed in a 250 mL flask and mixed with 80 mL of 2,2,4-trimethylpentane. Then, 5 mL of the mixed sample solution was added with 1 mL of ρ-anisidine solution, thoroughly mixed and left to react for 10 min in the dark. The absorbance was recorded at 350 nm using a UV-Vis spectrophotometer (GENESYS 180, Thermo Fisher Scientific, Bohemia, NY, USA). The PAV was calculated using Equation (6):

(6) $$\mathrm{PAV}=\frac{25\times \left(1.2\mathrm{As}-\mathrm{Ab}\right)}{\mathrm{weight}\;\mathrm{of}\;\mathrm{oil}\;\mathrm{or}\;\mathrm{fat}\;\mathrm{used}\;\mathrm{for}\;\mathrm{analysis}\;\left(g\right)}$$

where

As = the absorbance of the fat solution after reaction with ρ-anisidine reagent
Ab = the absorbance of the fat solution.

#### 2.5.4. Peroxide Value (PV)

The PV was measured following the AOAC Official Method 965.33 [33]. First, 1 g of oil sample (S) and blank (B) were prepared in two 250 mL Erlenmeyer flasks and 30 mL of a mixed solvent of chloroform-acetic acid (2:3 *v*/*v*) added. Then, 0.5 mL of saturated KI solution was added to each flask, shaken and allowed to stand in the dark for 1 min. The mixed samples were then immediately added to 30 mL of distilled water to stop the reaction. Finally, the mixture was titrated with 0.002 M sodium thiosulphate until the blue color disappeared. The result was expressed as peroxide milliequivalent per kg oil (meq/kg oil) using Equation (7):

(7) $$\mathrm{PV}\;\left(\mathrm{meq}/\mathrm{kg}\;\mathrm{samples}\right)=\frac{2\times \left(\mathrm{Samples}-\mathrm{Blank}\right)\mathrm{mL}\;}{\mathrm{weight}\;\mathrm{of}\;\mathrm{oil}\;\left(g\right)}$$

#### 2.5.5. Total Oxidation Value (TOTOX)

The overall primary and secondary oxidative state of the coffee oil was evaluated by calculating the TOTOX [25] using Equation (8):

(8) TOTOX = P−AV +2PV

#### 2.5.6. Measurement of Thiobarbituric Acid Reactive Substances (TBARS)

TBARS was measured following Rendon et al. [16]. First, 0.2 g of the ground coffee sample was added to 4 mL of 1% (*w*/*v*) trichloroacetic acid (TCA) containing 0.08 g of polyvinylpolypyrrolidone (PVPP) and the mixture was homogenized by continuous stirring for 30 min. Then, the mixture was centrifuged at 20,000 rpm for 10 min at 7 °C. Two milliliters of 20% TCA (*w*/*v*) solution was added, containing 0.5 mL of 0.5% (*w*/*v*) thiobarbituric acid of the supernatant. The reaction mixture was heated for 30 min in a water bath at 90 °C and cooled. The cooled mixture was then centrifuged at 10,000 rpm for 10 min at 10 °C. Quantification was performed by a spectrophotometer (Agilent Technologies, Santa Clara, CA, USA) at 532 and 600 nm using an extinction coefficient of 155 mM^−1^ cm^−1^, with results expressed as nmol of MDA/g of sample (d.w.).

#### 2.5.7. Fatty Acid Profile

The fatty acid profile was determined by gas chromatography-mass spectrometry (GC-MS). Ten milligrams of green coffee bean oil sample were placed in a 1.5 mL microcentrifuge tube and 500 μL of 0.5% (*w*/*v*) methanolic sodium hydroxide was added, followed by water bath shaking at 60 °C for 20 min. The sample was then cooled at room temperature, 1 mL of n-hexane was added and the mixture was vortexed for 1 min. Then 200 μL of distilled water was added and the mixture was vortexed for 30 s before centrifuging at 4500 rpm for 10 min at 25 °C. After centrifuging, the hexane layer was transferred into another tube and a small amount of anhydrous sodium sulfate was added to dry the residue water in the hexane before filtrating the fatty acids for analysis by GC-MS (6890N, Agilent Technologies, Santa Clara, CA, USA) [34,35] with an HP-5 column (0.25 mm × 30 m × 0.25 μm) and MS detector (5973N Agilent Technologies, Santa Clara, CA, USA). The temperature gradient was set at 150 °C for 10 min at 10 °C/min until reaching 250 °C and then 250 °C for 10 min. Helium was used as the carrier gas with a constant flow rate of 1 mL/min using injector split mode (150:1) at 220 °C and injector volume 1 μL.

### 2.6. Statistical Analysis

Lipid oxidation, physicochemical properties and oxidation results from different treatments were recorded as mean values ± standard deviations (*n* = 3). Statistical analysis was performed by analysis of variance (ANOVA) using SPSS statistical software (version 20, SPSS Inc., Chicago, IL, USA). Mean values were compared using Tukey’s test (*p* < 0.05) to determine significant differences between the treatments. Principal component analysis (PCA) and hierarchical clustering analysis (HCA) were performed using R Statistical Software (v4.1.2, R Core Team, Indianapolis, IN, USA, 2021) and results were normalized to exclude dimensional consequences before analysis.

## 3. Results and Discussion

### 3.1. Effect of Accelerated Storage on Change of GCB Qualities

**Figure 2:**
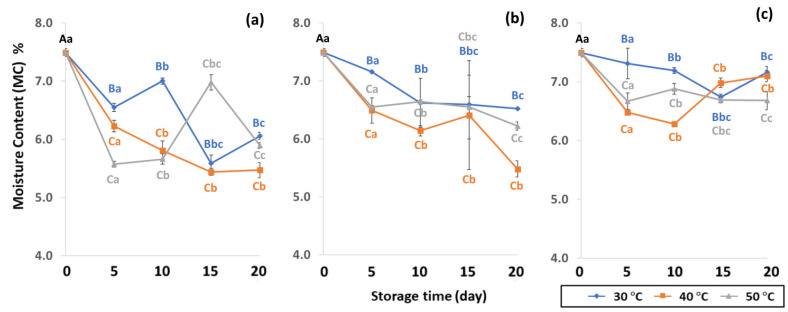
Effect of packaging on changes in moisture contents during accelerated storage conditions (**a**) PW, (**b**) LDPE and (**c**) GP. Different capital letters indicate significant differences among temperature at *p* < 0.05; different lower-case letters indicate significant differences among storage time at *p* < 0.05.

**Figure 3:**
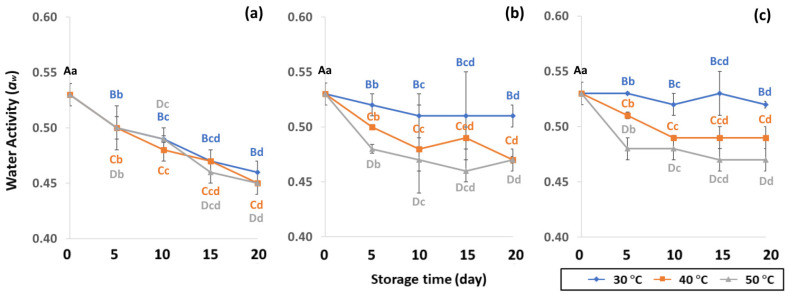
Effect of packaging on changes in water activity during accelerated storage conditions (**a**) PW, (**b**) LDPE and (**c**) GP. Different capital letters indicate significant differences among temperature at *p* < 0.05; different lower-case letters indicate significant differences among storage time at *p* < 0.05.

The moisture content (MC), water activity (*a*_w_) and color of GCBs under various packaging and accelerated storage conditions are shown in Figure 2 and Figure 3. Moisture content (MC), water activity (*a*_w_) and color were significantly different (*p* < 0.05) after accelerated storage in PW, LDPE and GP bags at 30 °C, 40 °C and 50 °C. The initial MC of GCB was 7.49 ± 0.19%. Storage time and temperature influenced the significant decrease of MC (*p* < 0.05). GCB stored at longer storage time with a higher temperature resulted in a more significant decrease of MC. Results revealed that GP bags maintained the MC of GCB better than PW and LDPE bags. According to Erdawati [36], GP bags adequately maintained GCB moisture content for one year and were suitable for GCB storage on a commercial scale under modified and controlled atmospheric conditions [11]. GP bags preserved GCB physical characteristics such as MC, shard, color and density, and also retained the contained compounds and chemical properties better than jute or plastic bags [11,14,15]. The MC of GCB is an important quality attribute that is regulated by coffee exporting and importing countries, long-term storage and the roasting process [37]. The optimal GCB moisture content is between 9% and 12% [38]. The initial MC content of green coffee beans was 7.49 ± 0.19% (w.b.), and lower than the safety range of 8 to 12.5% (w.b.) as the world coffee quality standard [38]. Low MC content in GCB causes an undesirable appearance and the beans shrink, leading to poor-quality coffee [37,39,40]. Lower MC with high unsaturated fatty acids in GCB forms oxidation reactions, producing rancid odors, loss of quality and secondary product oxidation [25]. A previous study reported MC ranging from 5.52 to 7.38% in GCB [41]. After 20 days of accelerated storage, MC reduced due to the high storage temperature of 50 °C, with a lower value compared to 30 °C and 40 °C. The GP bags were the best packaging for sustaining MC loss over LDPE and PW (Table S1). During the first 5, 10 and 15 days LDPE preserved the MC of GCB better than PW. PW bags allowed the free exchange of gases and water vapor between GCB and the ambient air and did not maintain specialty coffee quality [10]. Thus, packaging and storage conditions are related to lipid oxidation and have a direct impact on grain MC.

In the control samples, *a*_w_ content was 0.53 ± 0.01. Longer storage at 50 °C gave lower *a*_w_ content of PW (0.45 to 0.50), LDPE (0.47 to 0.48) and GP (0.47–0.48), while 30 °C retained higher *a*_w_ content for longer than at 40 °C and 50 °C. As a result, GCB in GP bags had a lower change in *a*_w_ content than beans packed in PW and LDPE bags. The lower storage temperature of 30 °C was preferable to 40 °C and 50 °C for maintaining *a*_w_ that is required to protect the GCB during the drying phase of processing and storage to achieve and maintain the coffee quality [42,43]. The *a*_w_ of GCB ranged from 0.45 to 0.53, and was similar to 0.45 to 0.55 as reported by Agudelo [44]. According to de Oliver et al. [45], the *a_w_* of GCB stored at 22 to 32 °C ranged between 0.95 and 0.99, which supported the growth of *A. carbonarius* and *A. chraceus*. Lower a_w_ content than 0.45 did not present enough water to maintain green coffee bean quality [43], while *a*_w_ content higher than 0.9 produced mold and fungi such as OTA [43,46]. As a consequence, *a_w_* is an important parameter for maintaining the physical properties of GCB during storage.

**Figure 4:**
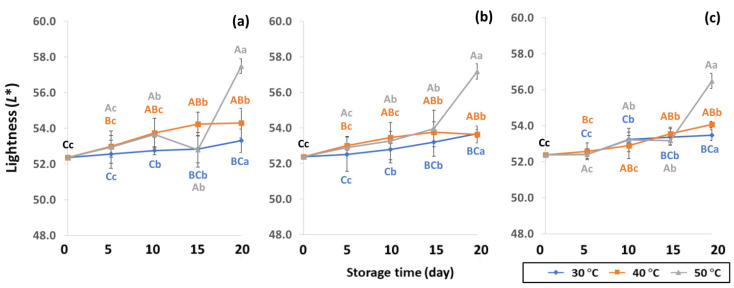
Effect of packaging on changes in lightness during accelerated storage conditions (**a**) PW, (**b**) LDPE and (**c**) GP. Different capital letters indicate significant differences among temperature at *p* < 0.05; different lower-case letters indicate significant differences among storage time at *p* < 0.05.

**Figure 5:**
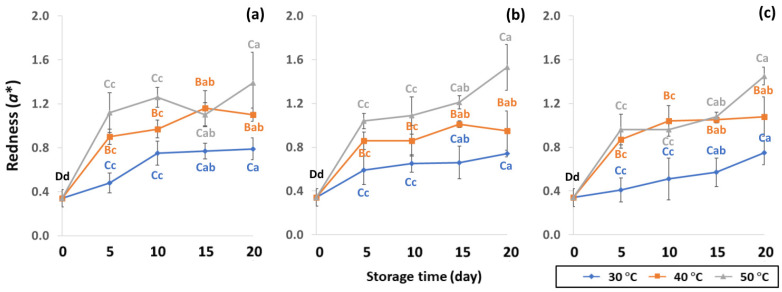
Effect of packaging on changes in redness during accelerated storage conditions (**a**) PW, (**b**) LDPE and (**c**) GP. Different capital letters indicate significant differences among temperature at *p* < 0.05; different lower-case letters indicate significant differences among storage time at *p* < 0.05.

**Figure 6:**
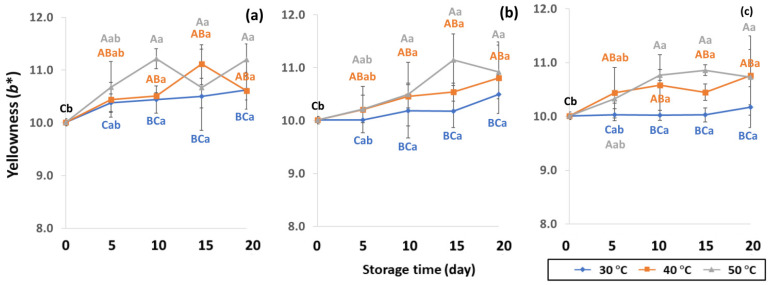
Effect of packaging on changes in yellowness during accelerated storage conditions (**a**) PW, (**b**) LDPE and (**c**) GP. Different capital letters indicate significant differences among temperature at *p* < 0.05; different lower-case letters indicate significant differences among storage time at *p* < 0.05.

GCB color is an important indicator of freshness, spoilage and homogeneity that influences coffee flavor [18,22]. Initial color values of the control GCB were 52.38 ± 0.07, 0.34 ± 0.08 and 10.01 ± 0.07 for L*, a* and b*, respectively (Figure 4, Figure 5 and Figure 6). Longer storage of 20 days increased the lightness of GCB, with GP bags showing less change than LDPE and PW bags. The highest a* value was recorded at 50 °C. Higher temperatures at longer storage contributed the most redness to GCB. Packaging types were not significantly different for changes of a* under the same storage conditions (Table S1). The b* values ranged from 10.1 ± 0.01 to 11.28 ± 0.22, with no significant differences under the same temperature conditions, while GCB color changed from bluish green to yellow. After 20 days of accelerated storage, GCB packed in LDPE and PW bags were brighter red and more yellow than those packed in GP bags. Faded GCB color resulted in a slightly bitter taste with woody or smoky notes [18,47]. GCB packed in GP bags was described as having a medium bright-greenish-bluish coloration as shiny, translucent and fresh [18,47,48]. The color of GCB is related to beverage quality, which strongly suggests that the oxidation process and natural enzymatic biochemical transformation are responsible for coffee flavor and aroma [15]. Longer storage at 50 °C showed a significant increase in L*, a* and b* compared to 30 °C and 40 °C. Higher temperatures of accelerated storage gave more brownish and yellowish coloration due to the Maillard reaction, as a strong indication of oxidation and enzymatic reaction. Moreover, after 20 days of storage at 50 °C, the silver skin on top of GCB broke up, making the bean whiter than in other accelerated storage conditions. The change in green coffee color during accelerated storage is related to low moisture content that results in lighter to whiter beans [11,49].

### 3.2. Effects of Accelerated Storage Conditions on GCB Lipid Oxidation

**Figure 7:**
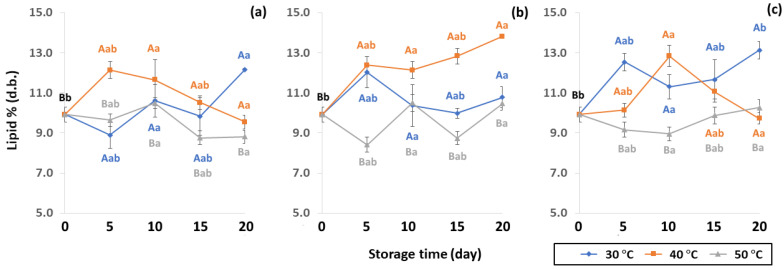
Effect of packaging on changes in lipid contents during accelerated storage conditions (**a**) PW, (**b**) LDPE and (**c**) GP. Different capital letters indicate significant differences among temperature at *p* < 0.05; different lower-case letters indicate significant differences among storage time at *p* < 0.05.

The chemical properties of lipid content were impacted by packaging during accelerated storage. Lipid contributes to beverage quality by influencing texture (crispness, plasticity and viscosity of liquid), mouthfeel (creamy, oily, richness, smoothness, chocolate, butter or baked), appearance (color and opacity) and flavor (aroma and taste, like fruit or vegetable flavors) [50]. Lipid content of GCB under accelerated storage ranged from 8.42 to 13.82% (d.b.). These results were in the same range as observed by Speer and Kolling-Speer [26] at 7 to 17%. Lipid content in GCB was also reported in the range of 10–15% [51,52]. Lipid content of GCB packed in PW, LDPE and GP bags was significantly different (*p* < 0.05), as shown in Figure 7. The initial value of lipid content in GCB was 9.92 ± 0.38% (d.b.). During accelerated storage, lipid content ranged from 8.76–12.18%, 8.42–13.82% and 8.96–13.14% for PW, LDPE and GP bags, respectively, with LDPE providing the highest lipid content. The increasing trend of lipid content at 40 °C of LDPE was observed. However, there was no previous report on the increase of the lipid content of GCB during storage. On the other hand, Hou and Chang [53] reported that lipid content of soybeans stored for nine months increased from 17.18% to 20.36%. The destruction of phospholipids during storage forms compounds that may become extractable in petroleum ether. Storage temperature at 50 °C showed lower lipid content, resulting in lipid oxidation causing product off-flavor, loss of aroma and taste [19,50]. Coffee lipid content is not only related to storage but is also impacted by particle size, surface area, choice of solvent and duration of extraction, processing, drying method and geographical origin [19,47,54,55]. Storage conditions and packaging types significantly influenced changes of GCB lipid oxidation of GCB (*p* < 0.05) (Table S2).

**Figure 8:**
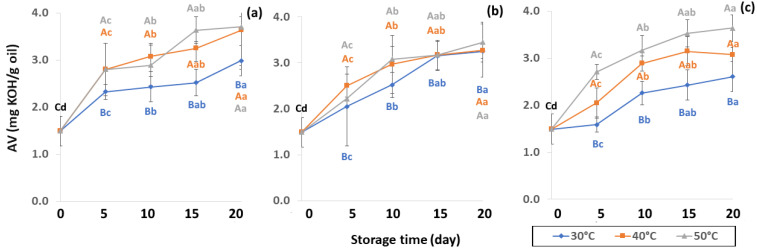
Effect of packaging on changes in acid value (AV) during accelerated storage conditions (**a**) PW, (**b**) LDPE and (**c**) GP. Different capital letters indicate significant differences among temperature at *p* < 0.05; different lower-case letters indicate significant differences among storage time at *p* < 0.05.

**Figure 9:**
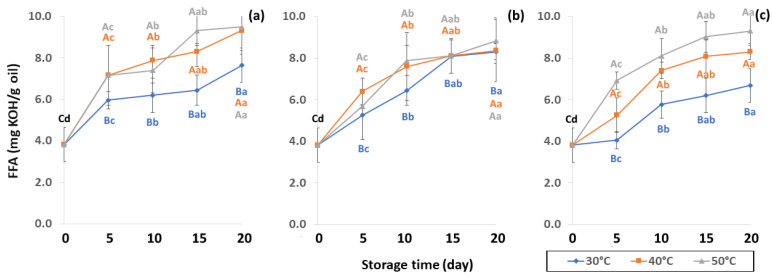
Effect of packaging on changes in free fatty acid (FFA) content during accelerated storage (**a**) PW, (**b**) LDPE and (**c**) GP. Different capital letters indicate significant differences among temperature at *p* < 0.05; different lower-case letters indicate significant differences among storage time at *p* < 0.05.

**Figure 10:**
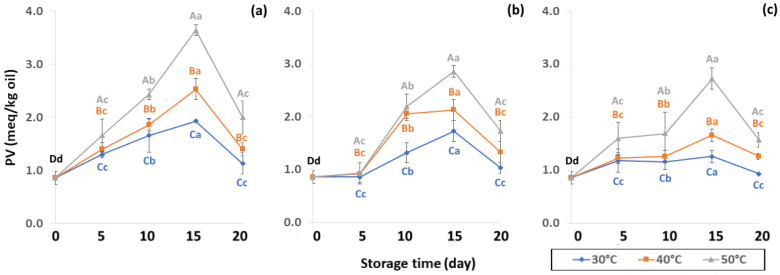
Effect of packaging on changes in peroxide value (PV) during accelerated storage (**a**) PW, (**b**) LDPE and (**c**) GP. Different capital letters indicate significant differences among temperature at *p* < 0.05; different lower-case letters indicate significant differences among storage time at *p* < 0.05.

Lipid oxidation, which is colorless and odorless, comprises the occurrence of primary oxidation, i.e., acid value (AV), free fatty acid (FFA), and peroxide value (PV), while secondary oxidation, i.e., ρ-anisidine value (PAV) and thiobarbituric acid reactive substances (TBARS) produce odors and off-flavors that decrease coffee quality. Initial values of primary oxidation in the control samples were AV (1.49 ± 0.32 mg KOH/g), FFA (3.82 ± 0.83 mg KOH/g), and PV (0.86 ± 0.12 meq/kg oil), as shown in Figure 8, Figure 9 and Figure 10. AV is defined as primary oxidation, which reflects total acidity and the number of fatty acids [56]. The AV of GCB significantly increased during storage for 20 days (*p* < 0.05). When GCB was conditioned at a higher temperature, AV and FFA levels rose faster. Arabica AV of GCB was higher than Robusta GCB at 0.79 to 1.28 [25], while AV was reported in various ranges of 3.89 mg KOH/g oil [57], 2.0 mg KOH/g [58] and 6.41 to 9.2 mg KOH/g [59].

FFA contents of GCB were reported at 3.57 to 10.48 mg KOH/g oil by Dong et al. [59] FFA content formed after hydrolysis degradation of lipid molecules is measured by AV to specify the degree of rancidity in oil hydrolysis [25]. Panpraneecharoen and Chumanee [60] considered PV and FFA as important parameters to determine the chemical quality of extracted coffee oil since both increased the oxidation reaction and reduced oil stability and degradation. Base-catalyzed esterification converts FFA extracted from oil into fatty acid methyl ester (FAME), an important parameter for maintaining product quality, with susceptibility to oxidation increased by high FFA [56]. Cong et al. [25] reported that FFA content can be used to measure the hydrolytic rancidity of triglycerides caused by enzymatic or spontaneous hydrolysis. FFA is also commonly used as an indicator of fat hydrolysis.

The PV increased until 15 days of storage (Figure 10) with an increase in hydrogen peroxide concentration. The high PV of coffee oil samples indicated low oxidative rancidity. The initial presence of free radicals in coffee promotes free radical formation associated with the pyrolysis reaction, therefore PV increases during storage as a result of lipid oxidation [61]. PV can be used as an oxidative index for the early stages of lipid oxidation with a slower increase to secondary oxidation. Initial PV concurred with Hong and Dong [62] who recorded 0.97 meq/kg of GCB. The PV increased until day 15 and was then affected by secondary oxidation, similar to results reported by Budryn et al. [63], where PV slowed down after nine weeks of regular storage. During the oxidation process, an abundance of primary products, peroxides and hydroperoxides are formed in oils via autoxidation [25]. PV is one of the most important initial products of autooxidation [64] that is related to oxidation caused by air at room temperature. Oxygen reacts with unsaturated fatty acids resulting in loss of flavor quality such as hay, grassy, green, fishy or sour as well as change in color and texture [64,65]. The PW bags showed faster lipid oxidation than LDPE and GP bags. The PV increased from 5 to 15 days and then decreased at 20 days, indicating the beginning of secondary oxidation. The GP bags gave a lower oxidation reaction than LDPE and PW bags (Table S2). Storage at high temperature of 50 °C accelerated the coffee lipid oxidation reaction.

**Figure 11:**
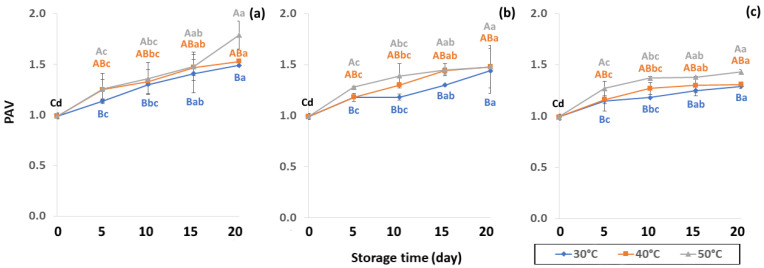
Effect of packaging on change ρ-anisidine value (PAV) during accelerated storage conditions (**a**) PW, (**b**) LDPE and (**c**) GP. Different capital letters indicate significant differences among temperature at *p* < 0.05; different lower-case letters indicate significant differences among storage time at *p* < 0.05.

**Figure 12:**
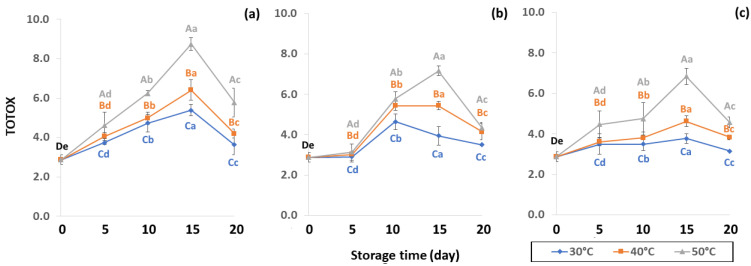
Effect of packaging on changes in TOTOX parameters during accelerated storage conditions (**a**) PW, (**b**) LDPE and (**c**) GP. Different capital letters indicate significant differences among temperature at *p* < 0.05; different lower-case letters indicate significant differences among storage time at *p* < 0.05.

**Figure 13:**
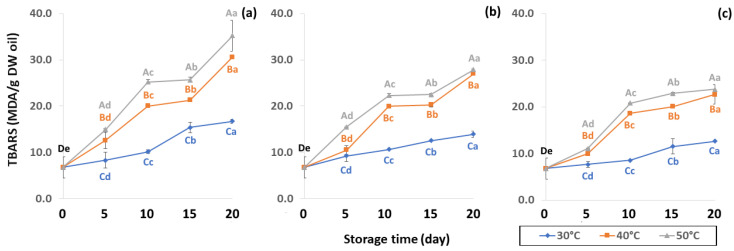
Effect of packaging on changes in TBARS during accelerated storage conditions (**a**) PW, (**b**) LDPE and (**c**) GP. Different capital letters indicate significant differences among temperature at *p* < 0.05; different lower-case letters indicate significant differences among storage time at *p* < 0.05.

Initial values of secondary oxidation in the control samples were 0.99 ± 0.03, 2.87 ± 0.24 and 6.76 ± 2.27 MDA/g DW for PAV, TOTOX and TBARS, as shown in Figure 11, Figure 12 and Figure 13. The PAV was similar to EI-Anany et al. [66] on roasted coffee oil (1.02). The initial TBARS value was also similar to Rendon et al. [16] at 8.8–10.2 MDA/g. PAV and TBARS are secondary terms for photooxidation and enzymatic oxidation that change relative to coffee flavor, taste, color and sensory characteristics [25,65,67]. PAV oxidation products arise from lipid decomposition by hydroperoxide to carbonyl, ketone and aldehyde compounds [67]. Acevedo et al. [68] suggested that PAV implies increased rancidity that impacted the quality, with increased oxidation of aldehydes and ketones in the end product. Results showed that greater amounts of secondary ethanol extract were generated at higher storage temperature. PAV contributes to the rancid flavor of oil, which can be detected by texture and mouthfeel. PAV with high concentration of secondary ethanol enhanced the oil flavor during storage [25], while TBARS increased significantly (*p* < 0.05) during accelerated storage (Figure 13). Dilnawaz et al. [69] found that increased TBARS was attributed to GCB extract containing many bioactive phytochemicals, polyphenols and flavonoids. TBARS are useful for determining secondary oxidation products that react with off-flavors such as ketones, esters, pyridines and other compounds. TBARS are malonaldehyde products that highly correlate to sensory scores [16,25]. To inhibit secondary oxidation, GP was shown to be the optimal packaging to maintain GCB quality. The TOTOX value includes data from primary and secondary oxidation analyses and indicates the overall oxidation stage of the oil under consideration [25]. The TOTOX value of GCB was calculated using PV and PAV. The change in TOTOX value followed the same trend as PV. According to Equation (8), the TOTOX values were approximately 2.5 times higher than those of PV. The maximum values of TOTOX were found to be 8.74 ± 0.33, 7.17 ± 0.25 and 6.84 ± 0.41 for PW, LDPE and GP, respectively, on 15 days under 50 °C. The TOTOX value gradually increased after storage and decreased when secondary oxidation began (Figure 12). TOTOX results showed that LDPE and GP bags had better capability to maintain GCB quality at the beginning of storage than PW. However, at a longer storage time, GP was preferable to prevent change in GCB quality.

### 3.3. Effects of Accelerated Storage Conditions on GCB Fatty Acid Profile

Changes in fatty acid composition under different accelerated storage conditions are shown in Table 2. GCB oil contained five major fatty acids as palmitic acid (C16:0), linoleic acid (C18:2), oleic acid (C18:1), stearic acid (C18:0) and arachidic acid (C20:0). Primary fatty acids of the control sample were C16:0 (35.64 ± 0.55%) and C18:2 (41.61± 0.60%), followed by C18:1 (10.00 ± 0.14%), C18:0 (7.25 ± 0.04%) and C20:0 (3.13 ± 0.02%). The main fatty acids presented in coffee oil were saturated fatty acids (palmitic acid C16:0, stearic acid C18:0 and arachidic acid C20:0) and unsaturated fatty acids (linoleic acid C18:2 and oleic acid C18:1). Palmitic acid and linoleic acid were the two most abundant acids in GCB, similar to reports by several previous studies [22,70,71,72].

Fatty acids are essential components of coffee flavor and aroma that influence coffee quality [73]. Unsaturated fatty acids (USFA), i.e., C18:2 and C18:1, were more prevalent than saturated fatty acids (SFA), including C16:0, C18:0 and C18:3. The amount of USFA decreased during storage, while SFA increased. Packaging type had no significant effect on change in total fatty acids (TFA) at the same storage time (Table S3). Longer storage caused a reduction in C18:0, C18:1 and C18:2, with an increase in C16:0 and C20:0 due to the oxidation reaction during accelerated storage. The high content of USFA provided less intense acidity, fragrance, body and flavor. Therefore, SFA including C16:0, C18:0 and C20:0 were likely discriminators of the sensory quality of specialty coffee [73,74]. High polyunsaturated fatty acids negatively impact final coffee quality because the double bonds are easily broken down, promoting the formation of undesired odor compounds [25].

### 3.4. Principle Component Analysis (PCA)

**Figure 14:**
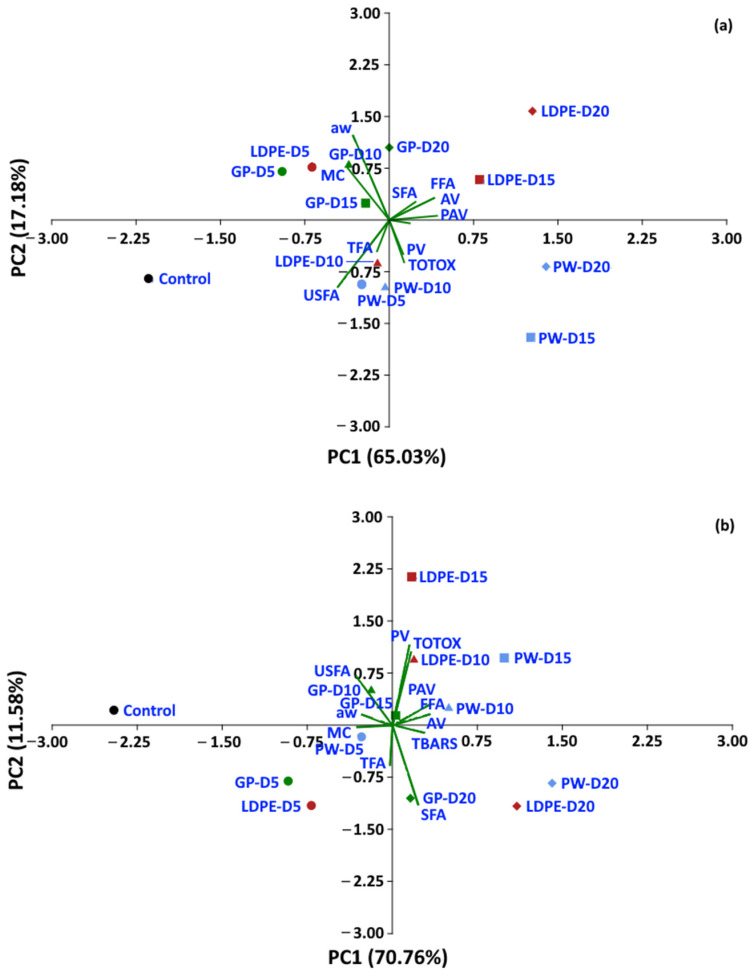

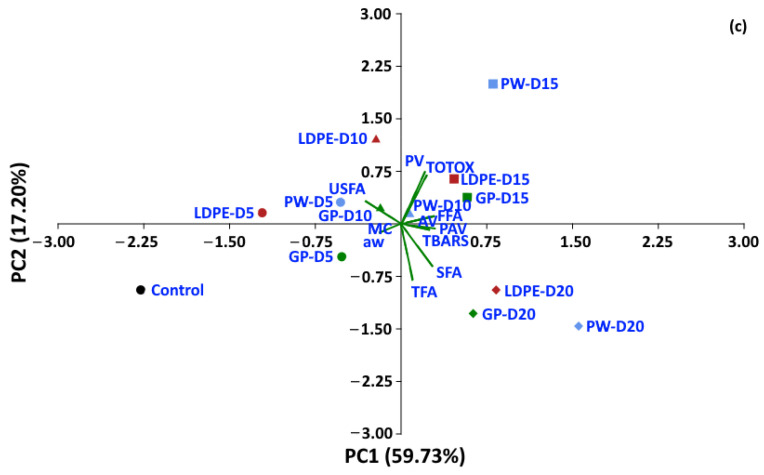
Biplot of the oxidation-related parameters of GCB under accelerated storage at (**a**) 30 °C, (**b**) 40 °C and (**c**) 50 °C. PW, plastic woven; LDPE, low-density polyethylene; GP, GrainPro^®^; *a*_w_, water activity; MC, moisture content; PV, peroxide value; AV, acid value; PAV, ρ-anisidine value; TBARS, thiobarbituric acid reactive substances; TOTOX, total oxidation value; TFA, total fatty acids; FFA, free fatty acids; SFA, saturated fatty acids; USFA, unsaturated fatty acids.

Statistical analyses were performed to determine whether the oxidative parameters of coffee could be discriminated between packaging type and storage period. A dataset of moisture content, *a*_w_ and oxidative parameters was used to perform PCA. For a storage temperature of 30 °C, the first two components of PCA explained 82.21% of the total variance (Figure 14). The control treatment is located on the left-hand side of the graphic, while storage values at 5 and 10 days are located at the center, and storage values for 15 and 20 days are located on the right-hand side of the graphic. GP packages were discriminated from other packages at the same storage time of 20 days (PC1 65.3%). For storage at 40 °C and 50 °C, the first two components of the PCA explained 82.34% and 76.93% of the total variance, respectively. The GP package was discriminated by PC1 (70.76%) from WP and LDPE. At 50 °C, GCB storage values at 5 and 10 days were distinguished from longer storage by PC1 (59.73%). The biplot (Figure 14a) also shows that FFA, PAV, SFA, TBARS, TOTOX and PV had positive loadings on PC1, while USFA, *a*_w_, MC and TFA had negative loadings on PC1. The loadings showed the same trend at higher storage temperatures of 40 °C and 50 °C. PV, TOTOX, PAV, AV, TBARS, SFA and TFA had positive loadings on PC1, while USFA, MC, and *a*_w_ had negative loadings. The PCA results showed that GCB data could be grouped based on storage time, while packaging type also influenced changes in the oxidative parameters. GCB packed in GP bags showed some minor overlap in the biplots with GCB packed in PW and LDPE bags at shorter storage times.

### 3.5. Agglomerative Hierarchical Clustering (AHC) Analysis

**Figure 15:**
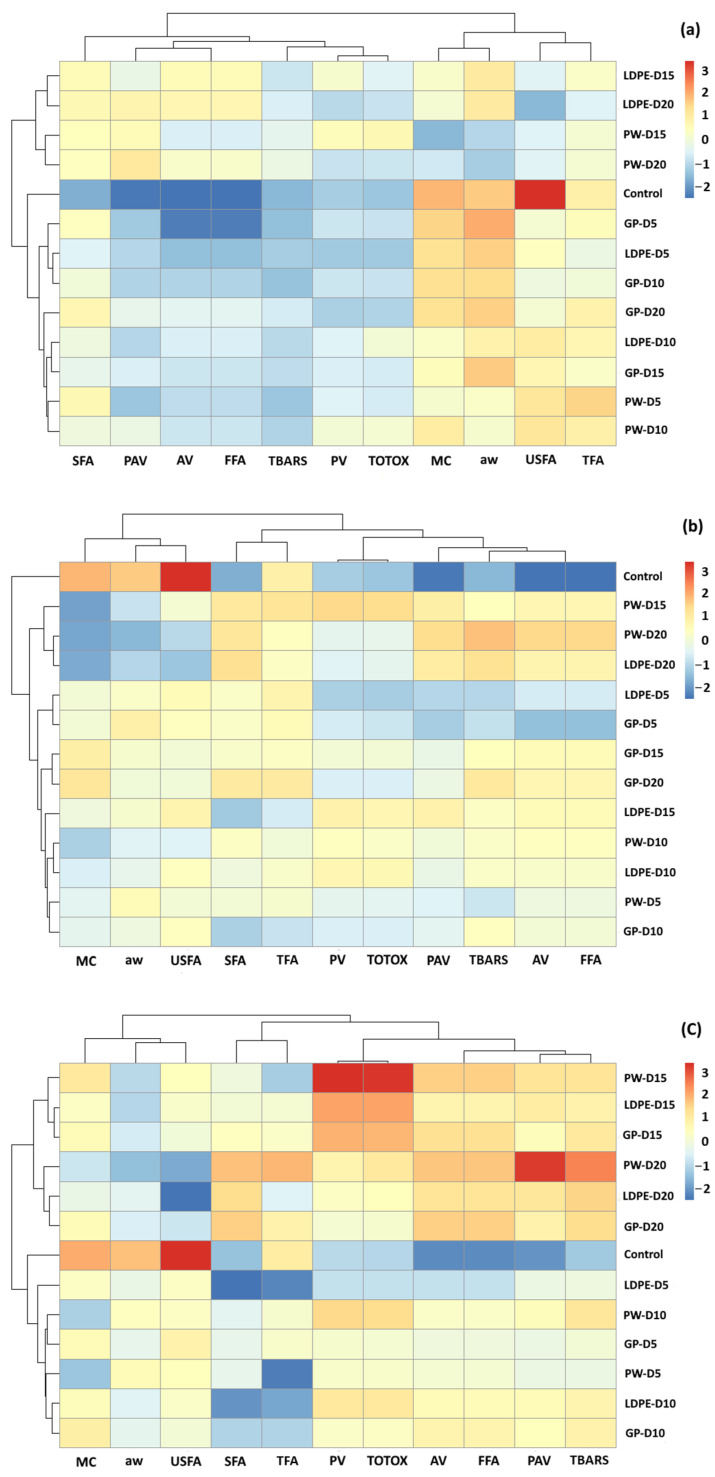
HCA of the oxidation-related parameters of GCB under accelerated storage at (**a**) 30 °C, (**b**) 40 °C and (**c**) 50 °C. PW, plastic woven; LDPE, low-density polyethylene; GP, GrainPro^®^; *a*_w_, water activity; MC, moisture content; PV, peroxide value; AV, acid value; PAV, ρ-anisidine value; TBARS, thiobarbituric acid reactive substances; TOTOX, total oxidation value; TFA, total fatty acids; FFA, free fatty acids; SFA, saturated fatty acids; USFA, unsaturated fatty acids.

According to the PCA results, overlaps were found for all storage temperatures (Figure 15). The effects of packaging influenced changes in GCB chemical components. Hierarchy clustering analysis (HCA) was performed to justify groups of GCB under different storage conditions. The clustering results were similar to those of the PCA biplot. At accelerated storage of 30 °C, GCB was grouped into three clusters. The first cluster was GCB with the control treatment, while the second cluster was GCB samples stored for 5 and 10 days with all packaging types. GCB packed in GP packages at 15 and 20 days were also classified into the second group. These results showed that the GP package delayed the change of oxidation reaction of GCB compared to PW and LDPE bags. The GCB stored at 15 and 20 days were classified into the third cluster, except for GP packaging. The control treatment for GCB stored at 5 to 10 days and GP packed at 15 and 20 days were characterized by MC, *a*_w_, USFA and TFA. GCB stored at longer storage times of 15 and 20 days were characterized by SFA, PAV, AV and FFA. At accelerated storage of 40 °C and 50 °C, clear clusters were formed between the control treatment and stored GCB. The GP packaging exhibited superior capability to delay the change of oxidative parameters. Moreover, oxidative parameters of PV, TOTOX, PAV and TBARS characterized GCB stored for longer time periods.

## 4. Conclusions

Changes in physical and chemical characteristics of GCB after undergoing accelerated storage with different packaging types were investigated. The different degrees of lipid oxidation were observed. Higher temperatures and longer storage time lead to a higher degree of lipid oxidation and quality change of GCB. PV, AV, PAV, TOTOX, TBARS, FFA and SFA values increased, while MC, *a*_w_ and USFA decreased during storage. PCA and HCA portrayed the influences of storage conditions and packaging types on change in GCB quality. Hermetic (GP) packaging was optimal for maintaining GCB quality, while selecting proper packaging delayed changes in GCB quality.

## Figures and Tables

**Table 1:** Properties of packaging materials.

Packaging	Air Permeability (L/m^2^ s)	Thickness (mm)	Surface Area (m^2^)	Supplier
PW	1054.62 ± 4.55	0.127 ± 0.00	0.0445	Siam Makro PCL, Bangkok, Thailand
LDPE	101.47 ± 2.70	0.075 ± 0.00	0.0445	Siam Makro PCL, Bangkok, Thailand
GP	0.538 ± 0.04	0.124 ± 0.00	0.0445	Intergro Co., Ltd., Bangkok, Thailand

**Table 2:** Effect of packaging on changes in fatty acids during accelerated storage conditions.

Fatty Acid (%)	Packaging	Control	30 °C, 50% RH				40 °C, 50% RH				50 °C, 50% RH			
		Day 0	Day 5	Day 10	Day 15	Day 20	Day 5	Day 10	Day 15	Day 20	Day 5	Day 10	Day 15	Day 20
**C16:0**	PW	35.64 ±0.55 Cc	35.41 ±0.02 Cc	35.10 ±0.22 Cb	35.44 ±0.13 Ca	36.35 ±0.05 Ca	36.35 ±0.13 BCa	35.38 ±0.03 BCa	35.76 ±0.03 BCa	37.31 ±0.58 BCa	35.88 ±0.05 Ac	37.23 ±0.13 Ab	36.99 ±0.25 Aa	37.84 34.84 Aa
	LDPE	35.65 ±0.06 Cc	35.53 ±0.08 Cc	36.06 ±0.37 Cb	35.18 ±0.04 Ca	35.66 ±0.71 Ca	35.21 ±0.22 BCa	35.19 ±0.71 BCa	35.83 ±0.21 BCa	37.49 ±0.36 BCa	36.04 ±0.04 Ac	36.81 ±0.41 Ab	37.76 ±0.08 Aa	36.79 ±0.07 Aa
	GP	35.66 ±0.06 Cc	35.31 ±0.04 Cc	35.12 ±0.04 Cb	35.60 ±0.06 Ca	35.19 ±0.05 Ca	35.76 ±0.04 BCa	37.32 ±0.19 BCa	37.11 ±0.01 BCa	37.01 ±0.04 BCa	37.60 ±0.02 Ac	37.01 ±0.04 Ab	37.23 ±0.05 Aa	37.24 ±018 Aa
**C18:0**	PW	7.25 ±0.04 Bbc	8.09 ±0.01 Ab	7.89 ±0.01 Ac	8.21 ±0.01 Ac	8.11 ±0.02 Aa	7.30 ±0.04 Bb	7.88 ±0.04 Bc	6.92 ±0.04 Bc	7.34 ±0.37 Ba	6.68 ±0.02 Cb	6.72 ±0.17 Cc	6.42 ±0.03 Cc	7.96 ±0.03 Ca
	LDPE	7.25 ±0.04 Bbc	7.70 ±0.02 Ab	7.56 ±0.08 Ac	8.21 ±0.15 Ac	8.01 ±0.03 Aa	7.96 ±0.07 Bb	7.90 ±0.25 Bc	7.27 ±0.06 Bc	7.37 ±0.37 Ba	6.69 ±0.02 Cb	6.25 ±0.06 Cc	6.53 ±0.01 Cc	7.37 ±0.13 Ca
	GP	7.25 ±0.04 Bbc	8.12 ±0.05 Ab	8.05 ±0.02 Ac	7.73 ±0.04 Ac	8.22 ±0.02 Aa	7.68 b ±0.01 Bb	6.44 ±0.06 Bc	7.07 ±0.02 Bc	7.50 ±0.50 Ba	6.47 ±0.01 Cb	6.45 ±0.02 Cc	6.85 ±0.05 Cc	6.97 ±0.07 Ca
**C18:1**	PW	10.00 ±0.14 Aa	10.07 ±0.07 ABbc	9.57 ±0.21 ABab	10.12 ±0.10 ABbc	9.76 ±0.04 ABc	9.58 ±0.22 Bbc	9.71 ±0.05 Bab	9.30 ±0.23 Bbc	8.82 ±0.29 Bc	8.87 ±0.06 Cbc	9.58 ±0.22 Cab	9.11 ±0.83 Cbc	8.95 ±0.46 Cc
	LDPE	10.00 ±0.14 Aa	9.49 ±0.03 ABbc	9.83 ±0.34 ABab	9.48 ±0.06 ABbc	9.45 ±0.11 ABc	9.35 ±0.11 Bbc	9.76 ±0.37 Bab	9.52 ±0.23 Bbc	9.09 ±0.14 Bc	8.97 ±0.06 Cbc	9.23 ±0.05 Cab	8.80 ±0.08 Cbc	8.85 ±0.73 Cc
	GP	10.00 ±0.14 Aa	9.70 ±0.06 ABbc	9.98 ±0.10 ABab	9.59 ±0.08 ABbc	9.84 ±0.51 ABc	9.86 ±0.02 Bbc	9.26 ±0.01 Bab	9.23 ±0.40 Bbc	8.71 ±0.02 Bc	8.55 ±0.05 Cbc	9.55 ±0.05 Cab	8.82 ±0.39 Cbc	9.03 ±0.65 Cc
**C18:2**	PW	41.61 ±0.60 Aa	40.64 ±0.56 Cbc	41.15 ±0.04 Cab	39.91 ±0.13 Cbc	40.28 ±0.56 Cc	40.69 ±0.31 BCbc	40.32 ±0.13 BCab	40.99 ±0.85 BCbc	41.00 ±0.04 BCc	41.39 ±0.35 Bbc	40.72 ±0.03 Bab	41.25 ±0.17 Bbc	40.44 ±0.04 Bc
	LDPE	41.61 ±0.60 Aa	40.93 ±0.02 Cbc	40.82 ±0.26 Cab	40.58 ±0.04 Cbc	40.13 ±0.10 Cc	41.13 ±0.10 BCbc	40.64 ±0.05 BCab	41.02 ±0.22 BCbc	40.61 ±0.04 BCc	41.33 ±0.02 Bbc	41.05 ±0.01 Bab	41.46 ±0.46 Bbc	40.23 ±0.12 Bc
	GP	41.61 ±0.60 Aa	40.58 ±0.04 Cbc	40.22 ±0.07 Cab	40.75 ±0.10 Cbc	40.70 ±0.10 Cc	40.56 ±0.09 BCbc	40.54 ±0.37 BCab	41.01 ±0.40 BCbc	41.01 ±0.01 BCc	41.80 ±0.01 Bbc	40.63 ±0.01 Bab	41.34 ±0.07 Bbc	40.79 ±0.15 Bc
**C20:0**	PW	3.13 ±0.02 Bb	3.79 ±0.01 Aa	3.58 ±0.02 Ab	3.90 ±0.01 Ab	3.62 ±0.02 Aa	3.33 ±0.06 Ba	3.89 ±0.01 Bb	2.87 ±0.01 Bb	2.92 ±0.18 Ba	2.75 ±0.03 Ca	2.90 ±0.13 Cb	2.53 ±0.03 Cb	2.97 ±0.03 Ca
	LDPE	3.13 ±0.02 Bb	3.42 ±0.01 Aa	3.28 ±0.12 Ab	3.89 ±0.03 Ab	3.63 ±0.01 Aa	3.93 ±0.07 Ba	3.82 ±0.20 Bb	3.15 ±0.11 Bb	2.81 ±0.15 Ba	2.69 ±0.10 Ca	2.61 ±0.05 Cb	2.57 ±0.01 Cb	3.49 ±0.41 Ca
	GP	3.13 ±0.02 Bb	3.73 ±0.01 Aa	3.78 ±0.02 Ab	3.47 ±0.01 Ab	3.92 ±0.02 Aa	2.55 ±0.02 Ba	2.91 ±0.02 Bb	3.00 ±0.01 Bb	2.60 ±0.03 Ba	2.75 ±0.01 Ca	2.98 ±0.05 Cb	2.98 ±0.05 Cb	3.51 ±0.47 Ca
**SFA**	PW	46.03 ±0.12 Bc	47.29 ±0.02 Ab	46.92 ±0.22 Aa	47.21 ±0.15 Aa	47.17 ±0.05 Aa	46.98 ±0.04 Ab	47.14 ±0.02 Ab	47.54 ±0.01 Ab	47.57 ±0.77 Aa	46.65 ±0.08 Ab	46.60 ±0.08 Ab	46.78 ±0.27 Ab	47.82 ±0.01 Aa
	LDPE	46.03 ±0.12 Bc	46.65 ±0.08 Ab	46.89 ±0.17 Aa	47.27 ±0.01 Aa	47.30 ±0.08 Aa	47.11 ±0.08 Ab	46.91 ±0.26 Ab	46.25 ±0.05 Ab	47.67 ±0.58 Aa	45.42 ±0.01 Ab	45.66 ±0.51 Ab	46.86 ±0.08 Ab	47.65 ±0.35 Aa
	GP	46.03 ±0.12 Bc	47.17 ±0.06 Ab	46.95 ±0.01 Aa	46.80 ±0.02 Aa	47.33 ±0.01 Aa	47.09 ±0.05 Ab	46.30 ±0.26 Ab	47.08 ±0.01 Ab	47.50 ±0.46 Aa	46.66 ±0.05 Ab	46.21 ±0.06 Ab	47.06 ±0.09 Ab	47.72 ±0.58 Aa
**USFA**	PW	51.61 ±0.74 Aa	50.71 ±0.49 Bb	50.71 ±0.17 Bb	50.03 ±0.04 Bb	50.04 ±0.52 Bc	50.27 ±0.53 Bb	50.03 ±0.08 Bb	50.29 ±0.80 Bb	49.83 ±0.25 Bc	50.36 ±0.41 Bb	50.31 ±0.25 Bb	50.36 ±0.66 Bb	49.39 ±0.50 Bc
	LDPE	51.61 ±0.74 Aa	50.41 ±0.04 Bb	50.65 ±0.08 Bb	50.06 ±0.03 Bb	49.58 ±0.06 Bc	50.48 ±0.06 Bb	50.40 ±0.01 Bb	50.54 ±0.33 Bb	49.70 ±0.01 Bc	50.30 ±0.10 Bb	50.28 ±0.18 Bb	50.26 ±0.04 Bb	49.07 ±0.84 Bc
	GP	51.61 ±0.74 Aa	50.28 ±0.02 Bb	50.20 ±0.01 Bb	50.53 ±0.01 Bb	50.29 ±0.01 Bc	50.40 ±0.60 Bb	50.40 ±0.36 Bb	50.27 ±0.41 Bb	50.23 ±0.40 Bc	50.51 ±0.08 Bb	50.18 ±0.05 Bb	50.16 ±0.32 Bb	49.82 ±0.50 Bc
**TFA**	PW	97.63 ±0.85 Aa	97.99 ±0.51 Ab	97.63 ±0.05 Ab	97.23 ±0.11 Aab	97.21 ±0.57 Aab	97.24 ±0.49 Ab	97.17 ±0.07 Ab	97.24 ±0.09 Aab	97.83 ±0.52 Aab	97.39 ±0.32 Bb	97.66 ±0.52 Bb	97.15 ±0.68 Bab	98.16 ±0.51 Bab
	LDPE	97.63 ±0.85 Aa	97.06 ±0.12 Ab	97.54 ±0.09 Ab	97.32 ±0.02 Aab	96.88 ±0.15 Aab	97.59 ±0.09 Ab	97.30 ±0.58 Ab	96.79 ±0.06 Aab	97.36 ±0.06 Aab	95.72 ±0.75 Bb	95.94 ±0.09 Bb	97.12 ±0.55 Bab	96.72 ±0.06 Bab
	GP	97.63 ±0.85 Aa	97.44 ±0.01 Ab	97.14 ±0.01 Ab	97.33 ±0.02 Aab	97.62 ±0.02 Aab	97.49 ±0.55 Ab	96.70 ±0.62 Ab	97.35 ±0.40 Aab	97.74 ±0.07 Aab	97.17 ±0.13 Bb	96.39 ±0.02 Bb	97.22 ±0.23 Bab	97.54 ±0.08 Bab

Note Data are presented as mean ± SD. Means with different capital letters in the same row indicate significant differences among temperature at *p* < 0.05; different lower-case letters in the same row indicate significant differences among storage time at *p* < 0.05. PW, plastic woven; LDPE, low-density polyethylene; GP, GrainPro^®^; RH, relative humidity; C16:0, palmitic acid; C18:0, stearic acid; C18:1, oleic acid; C18:2, linoleic acid; C20:0, arachidic acid; SFA, saturated fatty acids; USFA, unsaturated fatty acids; TFA, total fatty acids.

## Data Availability

The data are available from the corresponding author.

## Supplementary Materials

The following are available online at https://www.mdpi.com/article/10.3390/foods11193040/s1, Table S1: The effect of the type of packaging on physical properties, Table S2: The effect of the type of packaging on oxidation reaction, Table S3: The effect of the type of packaging on fatty acids.
