# Supplementary material for: Lipid Oxidation Changes of Arabica Green Coffee Beans during Accelerated Storage with Different Packaging Types
Source: Foods. 2022 Sep 30;11(19):3040. doi: 10.3390/foods11193040 (PMC9563479; doi:10.3390/foods11193040)
Supplement: Supplementary file 1 [file foods-11-03040-s001.zip › foods-1918553-supplementary.pdf]

**Table S1.** The effect of the type of packaging on physical properties

| Packaging | MC (%) | $a_w$ | *L** | *a** | *b** |
|---|---|---|---|---|---|
| Control | 7.49±0.07[a] | 0.53±0.01[a] | 52.38±0.07[b] | 0.34±0.08[b] | 10.01±0.07[b] |
| PW | 6.02±0.55[d] | 0.48±0.02[c] | 53.64±1.42[a] | 0.98±0.27[a] | 10.70±0.41[a] |
| LDPE | 6.45±0.46[c] | 0.49±0.02[b] | 53.62±1.30[a] | 0.93±0.28[a] | 10.47±0.47[a] |
| GP | 6.85±0.32[b] | 0.50±0.02[b] | 53.26±1.15[a] | 0.89±0.30[a] | 10.43±0.43[ab] |

**Note** Data are presented as mean ± SD. Means with different letters in the same column indicate significant differences at $p<0.05$. PW, plastic woven; LDPE, low-density polyethylene; GP, GrainPro®; MC moisture content, $a_w$, water activity; *L**, lightness; *a**, redness, and *b**, yellowness

**Table S2.** The effect of the type of packaging on oxidation reaction

| Packaging | Lipid | AV | FFA | PV | PAV | TOTOX | TBARS |
|---|---|---|---|---|---|---|---|
| Control | 9.92±0.38[b] | 1.49±0.32[b] | 3.82±0.83[b] | 0.86±0.12[c] | 0.99±0.03[b] | 2.87±0.24[c] | 6.76±2.28[d] |
| PW | 10.26±1.28[ab] | 3.01±0.58[a] | 7.70±1.48[a] | 1.91±0.69[a] | 1.40±0.19[a] | 5.21±1.45[a] | 19.70±8.09[a] |
| LDPE | 11.05±1.64[a] | 2.90±0.55[a] | 7.43±1.45[a] | 1.59±0.63[b] | 1.35±0.14[a] | 4.50±1.29[b] | 17.70±6.33[b] |
| GP | 10.90±1.61[a] | 2.77±0.64[a] | 7.10±1.42[a] | 1.46±0.47[b] | 1.34±0.20[a] | 4.20±1.00[b] | 15.86±6.00[c] |

**Note** Data are presented as mean ± SD. Means with different letters in the same column indicate significant differences at $p<0.05$. PW, plastic woven; LDPE, low-density polyethylene; GP, GrainPro®; AV, acid value; FFA, free fatty acid; PV, peroxide value; PAV, p-anisidine value; TOTOX, total oxidation value; and TBARS thiobarbituric acid reactive substances

**Table S3.** The effect of the type of packaging on fatty acids

| Packaging | C16:0 | C18:2 | C18:1 | C18:0 | C20:0 *ns* | SFA | USFA | TFA |
|---|---|---|---|---|---|---|---|---|
| **Control** | 35.65±0.06[c] | 41.61±0.60[a] | 10.00±0.14[a] | 7.25±0.04[b] | 3.13±0.02 | 46.03±0.12[b] | 51.61±0.74[a] | 97.63±0.85[a] |
| **PW** | 36.34±1.01[ab] | 40.74±0.54[b] | 9.45±0.50[b] | 7.49±0.63[a] | 3.25±0.48 | 47.14±0.57[a] | 50.20±0.55[b] | 97.24±0.81[ab] |
| **LDPE** | 36.13±0.90[b] | 40.83±0.41[b] | 9.31±0.39[b] | 7.40±0.62[ab] | 3.27±0.51 | 46.80±0.73[a] | 50.14±0.50[b] | 96.94±0.69[b] |
| **GP** | 36.46±0.94[a] | 40.83±0.43[b] | 9.44±0.48[b] | 7.30±0.66[b] | 3.23±0.49 | 46.99±0.47[a] | 50.27±0.32[b] | 97.26±0.44[ab] |

**Note** Data are presented as mean ± SD. Means with different letters in the same column indicate significant differences at $p<0.05$. PW, plastic woven; LDPE, low-density polyethylene; GP, GrainPro®; RH, relative humidity; C16:0, palmitic acid; C18:0, stearic acid; C18:1, oleic acid; C18:2, linoleic acid; C20:0, arachidic acid; SFA, saturated fatty acids; USFA, unsaturated fatty acids, TFA, total fatty acids, and *ns*, no significant
